# Post-transplant manifestation of ankylosing spondylitis: a case report and review of literature

**DOI:** 10.1186/s12882-021-02252-x

**Published:** 2021-01-31

**Authors:** Zawiasa-Bryszewska Anna, Brzezińska Olga, Kurnatowska Ilona, Makowska Joanna

**Affiliations:** 1 Department of Internal Diseases and Transplant Nephrology, The Medical University of Lodz, Lodz, Poland; 2 Department of Rheumatology, The Medical University of Lodz, Lodz, Poland

**Keywords:** Ankylosing spondylitis case report, Kidney transplantation, Chronic back pain, Immunosuppressive treatment

## Abstract

**Background:**

Ankylosing spondylitis (AS) is an insidiously progressive and debilitating form of arthritis involving the axial skeleton, characterized by chronic back pain and progressive spinal stiffness, and lessening of pain and stiffness with exercise. Due to subsequent manifestation in different organs, AS causes reduction in life expectancy, so early diagnosis and treatment are of great importance. No AS cases have been reported in solid-organ transplant recipients yet.

**Case presentation:**

A 58-year-old woman with end-stage renal disease due to chronic glomerulonephritis, after allogenic kidney transplantation 25 years earlier, with stable, good graft function, treated with chronic immunosuppressive therapy based on cyclosporine A, mycophenolate mofetil, and prednisone, with no previous history of a connective tissue disease presented fever up to 39 °C accompanied by pain localized in sacroiliac region radiating to the left lower limb. Detailed diagnostic procedures and x-rays of the lumbar spine and of the targeted sacroiliac joints revealed lesions characteristic of AS. Sulphasalazine was added to standard immunosuppression regimen with good clinical results.

**Conclusions:**

We report an adult kidney transplant recipient with a new onset of AS. The risk of relapse or new onset of inflammatory disease in transplant recipients is extremely low due to immunosuppressive therapy following transplantation. However, when it occurs, the clinical presentation is commonly atypical, often leading to delayed diagnosis.

## Background

The appearance of inflammatory and autoimmune diseases is quite low in patients after solid organ transplantation [1]. Adequate immunosuppressive therapy usually mitigates the risk of relapse or new onset of an autoimmune disease in this population [2]. The clinical presentation of inflammatory diseases in patients after transplantation is commonly atypical, often leading to delayed diagnosis.

Musculoskeletal pain is a common problem in kidney transplant (KTx) recipients, however, an acute inflammatory arthritis is rare. The differential diagnosis of joint and back pain is broad and includes septic arthritis, systemic infection, crystal arthropathies, autoimmune rheumatologic disorders, and medication-related adverse reactions [3].

Ankylosing spondylitis (AS), previously known as Bechterew’s disease, is a chronic, progressive inflammatory disease with a diverse clinical presentation [3]. AS is characterized by inflammation and new bone formation leading to fusion of the spine and sacroiliac joints. Chronic back pain, progressive spinal stiffness and improvement of pain and stiffness with exercise are the most common features of the disease [4]. Other musculoskeletal manifestations of AS include arthritis, enthesitis and dactylitis [5]. Clinical symptoms most frequently begin in late adolescence or in young adults, with 80% before the age of 30 and with a 3:1 to 2:1 male to female ratio [6, 7].

In addition to spinal inflammation, AS is characterized by a broad clinical spectrum of the extra-articular manifestations like uveitis, psoriasis, inflammatory bowel disease, or aortic insufficiency [8, 9] as well as an increased cardiovascular risk, several pulmonary, renal, and neurological complications or depression [10].

We present a case of late onset of seronegative spondyloarthropaty in KTX patient despite ongoing immunosuppressive treatment. The following description applies to become unusual both in terms of gender, age, comorbidities of the patient, as well as the fact that the patient was constantly treated with triple immunosuppressive regimen.

## Case presentation

A 58-year-old woman with end-stage renal disease caused by chronic glomerulonephritis, after allogenic KTx 25 years earlier, with stable, good graft function, treated with chronic immunosuppressive therapy based on cyclosporine A (CsA), mycophenolate mofetil (MMF), and prednisone, with no previous history of a connective tissue disease was admitted to the Nephrology Department with fever up to 39 °C accompanied by pain localized in the sacroiliac region radiating to the left lower limb of 2 weeks duration. Physical examination revealed nothing specific, her vital signs were stable. Initial laboratory data indicated an elevated C-reactive protein (CRP) concentration to 287 mg/l (normal range < 5 mg/l), white blood count (WBC) was 6.4 × 10 ^3^/μl, haemoglobin concentration 10.6 g/dl, serum procalcitonin was negative. Urinalysis showed proteinuria 0.1 g/l, and 15 to 20 leukocytes /higher-power field (HPF), with no erythrocytes in urinary sediment. The graft function was stable (serum creatinine concentration (sCr) 95 μmol/l with estimated glomerular filtration rate (eGFR_CKD-EPI_) - 57.2 ml/min/1.73m^2^), serum uric acid was 7.8 mg/dl (normal range 4.2–6.8 mg/dl), alanine and aspartate aminotransferase as well as bilirubin concentration were within normal limits. The blood level of CsA was in the range appropriate to the period after KTx. Hepatitis B, hepatitis C, HIV and CMV DNA tests were negative. Urine and blood culture were negative. Echocardiography was performed, but revealed nothing specific and endocarditis was excluded. The empiric antibiotic therapy with clindamycin was started. Nuclear magnetic resonance (NMR) of the lumbar spine was firstly described as inconclusive, although the patient complained of constant pain aggravation. On the tenth day of hospitalization oedema with a tenderness of the left knee appeared. Due to exudates and excessive warmth of the left knee, arthrocentesis was performed. Sixty millilitres of turbid fluid was withdrawn. The synovial fluid was positive for *Streptococcus sp. (saprophytic flora)* thus a prolonged, targeted vancomycin therapy was started (the patient was allergic to penicillin). Gout was excluded. Moreover, because of the resistant pain and oedema of the left lower limb, Doppler ultrasound was performed and showed active deep vein thrombosis. Low molecular weight heparin therapy was administered in therapeutic dose. A control ultrasound examination revealed no features of thrombosis or valvular insufficiency. Despite antibiotic treatment further high inflammation parameters were still noted (CRP 149.4 mg/l), thus further diagnosis for atypical infection was performed (the direct examination of synovial fluid for tuberculosis and in vitro QuantiFERON test, anti - Borrelia burgdorferi antibodies in class IgM and IgG – all negative). After failure of treatment and the patient’s continuing complaints, the patient was referred to the Department of Rheumatology.

At the time of admission to the Department of Rheumatology the patient reported left knee pain sustained for more than a month and lumbar spine pain, severity independent of physical exercise, exacerbating during the night and accompanied by morning stiffness. Joint mobility within the normal range, soreness during loading and maximum limbs bent. In addition, Patrick sign was positive on the left side. After more detailed anamnesis the patient confirmed occurrence of typical inflammatory back pain of nearly 20 years. The pain typically worsened with inactivity and improved with exercise and non-steroid anti-inflammatory drugs (NSAIDs) treatment. Morning stiffness was prolonged to nearly all day. Nocturnal pain was not awakening her. The patient’s spine motion was markedly limited in all directions: Otto’s test 2 cm, Schober’s test 1.5 cm, chest expansion 3.5 cm deepening the cervical lordosis. Bath Ankylosing Spondylitis Disease Activity Index (BASDAI) was 6.2 whereas Bath Ankylosing Spondylitis Functional Index (BASFI) 2.5. There was neither edema nor mobility limitations in other peripheral joints. No other significant deviations from the norm in the rest of physical examination were noted. The symptoms were accompanied by high erythrocyte sedimentation rate (ESR) 98 mm/h, CRP 119.8 mg/l following an increase of creatinine concentration up to 144 μmol/l (GFR_CKD-EPI_ 35.7 ml/min/1.73m^2^).

**Fig. 1:**
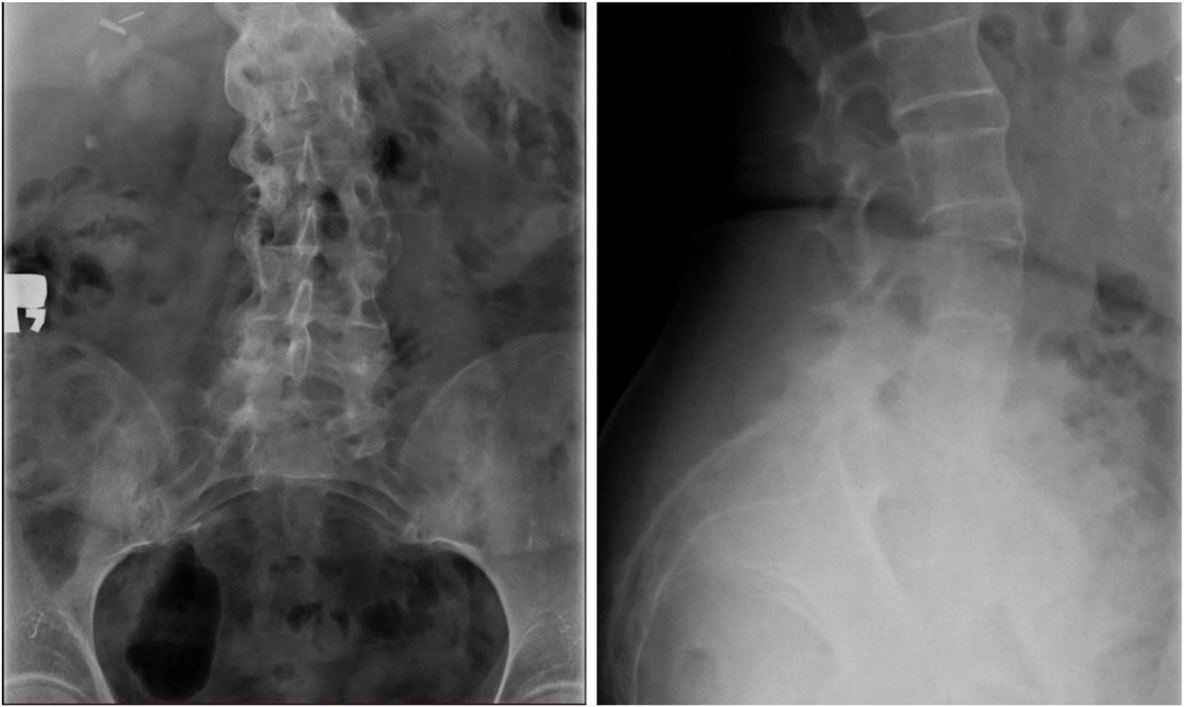
X-ray revealed diffuse syndesmophyitic ankylosis at all levels from Th12 to L1 (also seen above Th12), calcifications of spinal ligaments, features of vertebral body squaring, radiological signs of spondylodiscitis at levels from L1 to L4 with the widening of intervertebral space, blurring the trabecular structure of L5 and L6 narrowing of intervertebral spaces L4-S1. Left site scoliosis

During hospitalization the antibiotic therapy started in the Department of Nephrology was completed, whilst the anti-coagulant therapy was continued. An x-ray of the lumbar spine and of the targeted sacroiliac joints revealed lesions characteristic of ankylosing spondylitis: vertebral body squaring, diffuse syndesmophyitic ankyloses giving a “bamboo spine” appearance and total ankyloses of sacroiliac joints (Fig. 1). The patient was HLA B27 positive, autoantibodies like the antinuclear antibodies (ANA), anti- cyclic citrullinated peptides antibodies and rheumatoid factor were negative. After the negative result of control culture of the synovial fluid, intrarticular injection of bethametazone and lignocaine mixture was administered. The fluid fulfilled the criteria for the inflammatory fluid type I (Table 1). Uric acid crystals were not detected. The NMR of the lumbar spine done in the Nephrology Unit was assessed once again by a more experienced radiologist in the Rheumatology Unit and was described as typical features of ankylosing spondylitis. A diagnosis of ankylosing spondylitis was posed (Tables 2 and 3) and the patient was started on oral sulphasalazine and NSAIDs. The treatment resulted in a significant decrease of CRP and improvement of the graft function (sCr 92 μmol/l, eGFR 61.4 ml/min/1.73 m^2^). The diagnosis of chronic kidney disease (CKD) and KTx determined a reduction of sulphasalazine dose to 2x500mg and after a short therapy the NSAIDs administration was reduced. Radiosynovectomy in the left knee joint was performed and after 2 weeks the patient, in good general condition, was discharged home.

**Table 1:** Synovial fluid parameters

Synovial fluid parameters	
**Volume**	60 ml
**Colour**	Yellow
**Clarity**	Transparent
**Viscosity**	High
**Total protein**	50.3 g/l
**Glucose level**	Lower than blood
**WBC**	800/mm^3^
**PMNs**	> 50%
**Culture**	Negative

**Table 2:** Amor diagnostic criteria for spondyloarthropathy

Amor Criteria		Our patient
**Inflammatory back pain**	1 point	+
**Unilateral buttock pain**	1 point	–
**Alternating buttock pain**	2 point	–
**Enthesitis**	2 point	+/−
**Peripheral arthritis**	2 point	+
**Dactylitis (sausage digit)**	2 point	–
**Acute anterior uveitis**	2 point	–
***HLA-B27*** **–positive or family history of spondyloarthropathy**	2 point	+
**Good response to NSAIDs**	2 point	+
**Diagnosis of spondyloarthropathy with 6 or more points**		7–9 points

**Table 3:** New York diagnostic criteria for spondyloarthropathy

New York Criteria	Our patient
Low back pain with inflammatory characteristics	+
Limitation of lumbar spine motion in sagittal and frontal planes	+
Decreased chest expansion	+
Bilateral sacroiliitis grade 2 or higher	+
Unilateral sacroiliitis grade 3 or higher	
Definite ankylosing spondylitis when the fourth or fifth criterion mentioned presents with any clinical criteria	4 points

After 6 years of follow up during the routine visits in out-patient Transplantation Department the patient does not present any complaints associated with the left knee (no pain, tenderness, oedema). The graft function is still stable – creatinine concentration 90 μmol/l, GFR_CKD-EPI_ 62 ml/min/1.73m^2^, she has been treated long-term with her base immunosuppressive therapy (stable dose of CsA 75 mg bid, stable dose of MMF 500 mg bid, and temporarily, increased steroid dose up to 20 mg qd at the moment of AS diagnosis with the subsequent dose reduction to 5 mg qd), with reduced sulphasalazine dose (2 × 500 mg), with no adverse effects of such treatment.

## Discussion and conclusion

Joint pain is a frequent problem in individuals with kidney disease and is common both before and after KTx [11]. However, kidney involvement can be one of the complications following rheumatic diseases and can occur as a secondary amyloidosis which is the most common cause of nephrotic syndrome in AS followed by IgA nephropathy, mesangioproliferative glomerulonephritis as well as, rarely, by membranous nephropathy or focal segmental glomerulosclerosis [10]. A study by Hill et al. showed an 18% prevalence of CKD stages 3 to 5 in the rheumatic outpatient group, as compared to 5% reported within the general population [12]. On the other hand, it was shown that rheumatic diseases were the cause of ESRD and KTx in 14% of recipients transplanted in Poland between 1998 and 2015 years [13]. The authors showed that patients and graft survival were distinctly better in nonrheumatic recipients in comparison with rheumatic patients [13].

Differential diagnosis of joint and back pain in patients after successful KTx should take into account common complications of dialysis including crystal deposition disease, β_2_- microglobulin amyloidosis, secondary hyperparathyroidism, or aluminium overload as well as articular complications followed by hepatitis B and C or a variety of other infections [14]. In KTx recipients some new musculoskeletal and articular disorders, like infection-related arthralgia, polyarticular gout, rheumatoid arthritis flare, or a medication-related adverse reaction, namely steroid induced osteopenia or osteoporosis, may occur [11].

To the best of our knowledge, the present work is the first case report of late onset of seronegative spondyloarthropathy in a transplanted patient despite ongoing immunosuppressive treatment. De novo seropositive erosive rheumatoid arthritis in a patient 7 years after KTx was described by Forslund et al. in 2005 [15].

Immunosuppressive treatment after transplantation on the one hand prevents rejection of the transplanted organ, and on the other hand is an excellent treatment for diseases of the autoimmune and inflammatory background, preventing an exaggerated immune response. However certain injuries may alter this balance leading to dysregulation of the intestinal immune environment promoting the development of chronic inflammatory disease [16]. Immunosuppression heightens susceptibility to infectious agents that may damage the epithelial barrier of the intestinal mucosa resulting in prolonged exposure to luminal antigens followed by chronic immune stimulation [16].

Calcineurin inhibitors including CsA and tacrolimus strongly inhibit production of interleukin-2 (IL-2), which is normally produced by T cells to induce expansion of antigen-specific clones in response to a previously encountered antigen. Deficiency of IL-2 can result in T-cell dysregulation, leading to the development of chronic inflammation [17]. Arthralgia, muscular side effects and osteoarthropathy are some of the described side effects of CsA associated therapy. It was shown that high CsA levels (> 200 ng/ml) are the risk factors of joint pain in KTx recipients [11]. Our patient had the CsA trough level low, appropriate to time passed after KTx.

It has been found that mycophenolate mofetil can be associated with an acute inflammatory response characterized by fever, arthralgia, oligoarthritis and raised inflammatory markers appearing 3 to 5 days after initiation of therapy with MMF. Symptoms rapidly resolve with mycophenolate cessation. The pathogenesis has been associated with paradoxical pro-inflammatory reaction of polymorphonuclear neutrophils [18]. Our patient was treated with MMF for years, thus it is unlikely to have been the cause of symptoms.

The appearance of inflammatory disease regardless of adequate immunosuppressive therapy in transplant recipients is not unusual or impossible, however there is no data available associated with new onset of AS after transplantation. Moreover the most typical risk group of AS are young men, whereas the presented patient is a 58 year old woman. Very rarely does AS begin after the age of 40 resulting in difficulties in making the diagnosis, since standard classification scales are not validated for patients above 45 years old [19, 20].

The diagnosis of arthritis is primarily clinical. The most typical clinical features of arthritis of native joints are acute joint pain, swelling, warmth, erythema, decreased range of motion, fever, and general malaise. AS is characterised by the presence of spinal pain, resulting in limitation of spinal mobility reported also by our patient. These symptoms can be supported by laboratory evidence like leukocytosis or inflammatory markers including CRP, procalcitonin and ESR, which can be useful especially in patients with native joints without underlying haematological or rheumatological conditions. However, all of these laboratory tests have quite low sensitivity and specificity. Laboratory tests are usually followed by X-ray, ultrasound, computer tomography (CT) or magnetic resonance imaging (MRI) of the involved joint. To diagnose joint infection aspiration of the joints can be performed, however mainly in large joints.

As written above the immunosuppressive treatment can be associated with increased risk of infectious diseases. Therefore, the most common cause of monoarticular arthritis in both immunocompetent and immunocompromised patients is septic arthritis [21]. Symptoms of AS (low back pain) were reported by our patient for 20 years, but as low back pain is a common condition and the patient did not present other features of spondyloarthropathy like enthesitis, dactylitis, uveitis, diarrhoea or family clustering, the patient was not referred to a rheumatologist and the complaints were described as non-specific, mechanical low back pain. It cannot be excluded that the symptoms of AS were present earlier but were masked by anti-inflammatory properties of immunosuppressive drugs.

Although most cases of viral and reactive arthritis are connected with infectious background, they are usually not categorized as septic arthritis [21]. Monoarticular arthritis can result from viral infection caused by parvovirus B19, HIV or rubella, as well as from the deposition of immune complexes and complement components in hepatitis B and C infections [19]. In our patient the viral infections were excluded.

Reactive arthritis usually arises from an acute infection of the genitourinary or gastrointestinal tract caused by *Shigella spp., Salmonella spp., Campylobacter jejuni*, or *Yersinia enterocolitica*. It may also be one of the manifestations of Lyme disease [21, 22].

In the traditional understanding of septic arthritis the most commonly implicated organisms causing bacteremic septic arthritis are *Staphylococcus aureus*, *Streptococcus pyogenes, Neisseria meningitides, Neisseria gonorrhoeae, Klebsiella pneumonia, Escherichia coli, Proteus spp., Salmonella spp., Morganella morganii, Citrobacter spp., Serratias spp* or *Pseudomonas aerginosa* [22–24]. Septic arthritis can also result from mycobacteria (*Mycobacterium tuberculosis*), or fungi infections (*Histoplasma capsulatum, Blastomyces dermatitidis, Coccidioides immitis*) [21].

The most common predisposing factors for septic arthritis include age greater than 80 years, diabetes mellitus, rheumatoid arthritis, prosthetic joint, recent joint surgery, skin infection and skin ulcers, intravenous drug abuse and alcoholism, and previous intra-articular corticosteroid administration [25].

Transplant patients are particularly susceptible to develop septic arthritis caused by unusual organisms. These infections are commonly disseminated, involving both the bones and joints [25]. In the first few weeks after transplantation, joint infections are often hematogenous and caused by health-care-associated pathogens such as *Staphylococcus aureus* (including MRSA) and gram-negative bacilli like *Pseudomonas aeruginosa* [26]. However, in the months following transplantation, as patients are maintained on immunosuppressive therapy, they are more prone to infections caused by fungi and mycobacteria [26]. In our recipients fungi and mycobacteria were excluded.

Gout etiology was thought unlikely in our patient as no crystals were present on joint aspirate and the patient had no history of gout, with only slight elevated serum uric acid concentration [19] – thus this diagnosis was excluded in our case.

Following the modified New York criteria for AS it has been shown that radiographic sacroilitis is a rather late finding in the disease course of many patients [3]. The most typical radiological evidence for AS are structural changes in the sacroiliac joints and the spine. In our patient the typical features like vertebral body squaring, diffuse syndesmophyitic ankyloses giving a “bamboo spine” appearance and total ankyloses of sacroiliac joints were described on X-ray, whereas MRI revealed nothing specific.

Progression of AS varies among individuals, but some general trends can be observed. Younger age (≤40 years) at disease onset is usually associated with a predominance of axial symptoms, whereas patients with a later disease onset tend to present more peripheral manifestations [9]. Symptoms presented by our patient were associated with the spinal column despite her belonging to an older group.

The main purpose of treating the patient with AS is to improve long-term health-related quality of life by controlling the symptoms of the disease and averting continuous structural damage, thus leading to better joint function followed by improved social participation [3]. The treatment of AS should be personalised according to the present symptoms of the disease, patient’s comorbidities, possible complications resulting from the treatment and psychosocial factors [3].

The first-line drug treatment for patients complaining of pain and stiffness are NSAIDs administered up to the maximum dose, taking into consideration risks and benefits resulting from the treatment [27, 28]. Data from some studies revealed that in young patients failure to administered NSAIDs is associated with an increased mortality [29, 30]. For patients with good response to NSAIDs continuous use is recommended [3]. This first-line treatment should be enhanced by physical therapy including home exercises and by smoking cessation [31]. In patients who are contraindicated to NSAIDs or poorly tolerate this treatment, analgesics such as paracetamol and opioid-(like) drugs should be considered [3].

Lack of efficacy or presence of toxicity with NSAIDs is an indication to consider alternative treatments either with glucocorticoid, sulfasalazine or biological disease-modifying antirheumatic drugs (bDMARDs) [3].

Steroid injections directed to the local site of musculoskeletal inflammation may be recommended in patients with predominant peripheral manifestation as an option to treat arthritis and enthesitis, although direct evidence is lacking. Patients with axial disease should not be treated with systemic glucocorticoids for a long time [3].

Sulfasalazine may be considered in patients with peripheral arthritis, however it does not alleviate axial symptoms [32].

High disease activity defined as BASDAI ≥4 despite conventional treatment, elevated CRP, presence of inflammation on MRI of the SI joints and/or spine or presence of radiographic sacroiliitis is an indication to start bDMARDs and current practice is to start with tumour necrosis factor inhibitor (TNFi) therapy [31].

Our patient due to KTx was put on immunosuppressive therapy including CsA, MMF and steroids. Given the ongoing severity of the patient’s symptoms, no response to maintenance immunosuppressive therapy, contraindication to NSAIDs, a change in management was instituted by the administration of sulfasalazine. Improvement in the patient’s inflammatory markers and arthritis was noticed with subsidence of pain complaints and normalization of CRP within 2 weeks. She has had no further recurrence of acute inflammatory arthritis.

The establishing of proper diagnosis and management of a patient after KTx is difficult. Despite long-term immunosuppressive treatment in solid organ transplant recipients, in the differential diagnosis of joint pain the development of de novo arthritis should be considered, and when conclusive diagnosis is made, the patient may require administration of additional immunosuppressive drugs.

## Data Availability

The patient results used and/or analysed during the current case report are available from the corresponding author on reasonable request.

## Abbreviations

ANA: Antinuclear antibodies
anti-CCP: Anti- cyclic citrullinated peptides
AS: Ankylosing spondylitis
BASDAI: Bath Ankylosing Spondylitis Disease Activity Index
BASFI: Bath Ankylosing Spondylitis Functional Index
bDMARDs: Biological disease-modifying antirheumatic drugs
CKD: Chronic kidney disease
CRP: C-reactive protein
CsA: Cyclosporine A
CT: Computer tomography
eGFR: Estimated glomerular filtration rate
ESR: Erythrocyte sedimentation rate
ESRD: End-stage renal disease
HPF: Higher-power field
Ktx: Kidney transplantation
MMF: Mycophenolate mofetil
MRI: Magnetic resonance imaging
NMR: Nuclear magnetic resonance
NSAIDs: Non-steroid anti-inflammatory drugs
SCr: Serum creatinine concentration
TNFi: Tumour necrosis factor inhibitor
WBC: White blood count
