# Correction to: Post-transplant manifestation of ankylosing spondylitis: a case report and review of literature

**DOI:** 10.1186/s12882-021-02280-7

**Published:** 2021-03-03

**Authors:** Anna Zawiasa-Bryszewska, Olga Brzezińska, Ilona Kurnatowska, Joanna Makowska

**Affiliations:** 1 Department of Internal Diseases and Transplant Nephrology, The Medical University of Lodz, Lodz, Poland; 2 Department of Rheumatology, The Medical University of Lodz, Lodz, Poland

**Correction to: BMC Nephrol (2021) 22:46**

**https://doi.org/10.1186/s12882-021-02252-x**

Following publication of the original article [1], the authors informed us that the given name and family name of all the authors were switched.

The author group has been updated above and the original article [1] has been corrected.
